# GRK3 is a direct target of CREB activation and regulates neuroendocrine differentiation of prostate cancer cells

**DOI:** 10.18632/oncotarget.9359

**Published:** 2016-05-14

**Authors:** Meixiang Sang, Mohit Hulsurkar, Xiaochong Zhang, Haiping Song, Dayong Zheng, Yan Zhang, Min Li, Jianming Xu, Songlin Zhang, Michael Ittmann, Wenliang Li

**Affiliations:** ^1^ Texas Therapeutics Institute, Brown Foundation Institute of Molecular Medicine, University of Texas Health Science Center at Houston, Houston, TX, USA; ^2^ Department of Pathology and Laboratory Medicine, University of Texas Health Science Center at Houston, Houston, TX, USA; ^3^ Department of Molecular and Cell Biology, Baylor College of Medicine, Houston, TX, USA; ^4^ Department of Pathology and Immunology, Baylor College of Medicine, and Michael E. DeBakey VAMC, Houston, TX, USA; ^5^ Graduate School of Biomedical Sciences, University of Texas Health Science Center at Houston, Houston, TX, USA; ^6^ Division of Oncology, Department of Internal Medicine, and Memorial Herman Cancer Center, University of Texas Health Science Center at Houston, Houston, TX, USA; ^7^ Tumor Research Institute, the Fourth Hospital of Hebei Medical University, Shijiazhuang, China; ^8^ Breast and Thyroid surgery center, The Union Hospital of Tongji Medical College, Huazhong University of science and technology, Wuhan, China; ^9^ Department of Medical Oncology, Nanfang Hospital, Southern Medical University, Guangzhou, China; ^10^ The Union Hospital of Tongji Medical College, Huazhong University of science and technology, Wuhan, China

**Keywords:** GRK3, CREB, androgen deprivation therapy, neuroendocrine prostate cancer

## Abstract

Neuroendocrine prostate cancer (NEPC) is an aggressive subtype of prostate cancer that commonly arises through neuroendocrine differentiation (NED) of prostate adenocarcinoma (PAC) after therapy, such as radiation therapy and androgen deprivation treatment (ADT). No effective therapeutic is available for NEPC and its molecular mechanisms remain poorly understood. We have reported that G protein-coupled receptor kinase 3 (GRK3, also called ADRBK2) promotes prostate cancer progression. In this study, we demonstrate that the ADT-activated cAMP response element binding protein (CREB) directly targets and induces GRK3. We show GRK3 expression is higher in NEPC than in PAC cells and mouse models, and it positively correlates with the expression and activity of CREB in human prostate cancers. Notably, overexpression of GRK3 in PAC cells increased the expression of NE markers in a kinase activity dependent manner. Conversely, silencing GRK3 blocked CREB-induced NED in PAC cells, reversed NE phenotypes and inhibited proliferation of NEPC cells. Taken together, these results indicate that GRK3 is a new critical activator of NE phenotypes and mediator of CREB activation in promoting NED of prostate cancer cells.

## INTRODUCTION

Progression to castration resistant prostate cancers (CRPC) is a major therapeutic challenge for prostate cancer patients. Unfortunately, the mechanisms underlying CRPC development remain largely unclear. Approximately 25% of men who die of prostate cancer have tumors with a neuroendocrine phenotype [1–4]. Neuroendocrine prostate cancer (NEPC) is characterized by loss of androgen receptor (AR) expression, resistance to hormonal therapies, and elevated levels of NE-related proteins, such as enolase 2 (neuronal, ENO2) and chromogranin A and B (CHGA/CHGB) [1–4]. NEPC is associated with aggressive disease, frequent metastases to soft tissues and a short survival time [5–9]. With the recent introduction of potent ADT drugs, such as enzalutamide and abiraterone acetate, the incidence of NEPC is expected to increase dramatically [1, 2, 10–14]. A better understanding of the molecular events underlying NEPC development is urgently needed to develop a therapeutic solution for CRPC/NEPC.

NEPC can arise *de novo*, but most commonly evolves from preexisting prostate adenocarcinoma (PAC) [15–18]. The majority of evidence to date favor a transdifferentiation model of NEPC origin, where PAC treated extensively with androgen deprivation therapy (ADT) or radiation therapy develop into NEPC, as a mechanism of adaptive response and drug resistance [8, 9, 16–31]. Recently, neuroendocrine differentiation (NED) has been observed in a patient-derived xenograft model of prostate adenocarcinomas that developed NEPC after medical castration [14, 32, 33]. Many NED-inducing stimuli (such as androgen depletion and irradiation) act through increasing the intracellular level of cAMP that activates protein kinase A (PKA), which in turn activates CREB (cAMP response element-binding protein) via phosphorylation at S133 [15, 21, 23, 24, 28, 34–36]. Activated PKA is sufficient to induce neuroendocrine-like differentiation of LNCaP cells, while suppressing CREB activation through expressing CREB shRNA or a dominant negative inhibitor of CREB (A-CREB) has been shown to inhibit irradiation-induced NED in LNCaP cells [15, 21, 23, 24, 28, 34–36]. However, it remains incompletely understood how CREB activation induces NED in prostate cancer cells.

Through unbiased shRNA and cDNA screening of hundreds of human kinases, we have shown that G-protein coupled receptor kinase 3 (GRK3) is a new critical activator of prostate cancer progression [37]. Not only is it necessary for the survival and proliferation of metastatic cancer cells *in vitro* and *in vivo*, but it is also sufficient to promote primary tumor growth in the prostate and metastases in soft tissues (lungs and lymph nodes). Notably, GRK3 is overexpressed in human prostate tumors, especially in soft tissue metastases [37]. However, it is unknown what biological processes are responsible for GRK3 overexpression in prostate cancers and how GRK3 contributes to prostate cancer progression.

GRK3 belongs to the subfamily of G-protein coupled receptor kinases (GRKs). GRKs are best known for their roles in the phosphorylation and desensitization of agonist-bound GPCRs [38–42], including beta-adrenergic receptors (ADRBs). ADRBs act through the increase of cAMP by adenylyl cyclase (AC) and activation of PKA/CREB pathway [43–45]. Therefore, PKA/CREB can be activated through ADRB stimulation (such as isoproterenol) [43], or by a direct activator of AC, forskolin (FSK) and the inhibitor of phosphodiesterase, IBMX [46]. To understand how GRK3 and NEPC progression contribute to poor prognosis in prostate cancer, we investigated the mechanisms of GRK3 overexpression in prostate cancer and its connections to ADT, CREB activation and NEPC development. We show that GRK3 indeed controls NED phenotypes of prostate cancer cells, and is induced by ADT as a direct target and critical mediator of CREB activation. These results elucidate the mechanisms of NED in prostate cancer cells and may facilitate establishment of GRK3 as a new therapeutic target for NEPC.

## RESULTS

### ADT induces neuroendocrine differentiation of human prostate adenocarcinoma LNCaP cells

**Figure 1:**
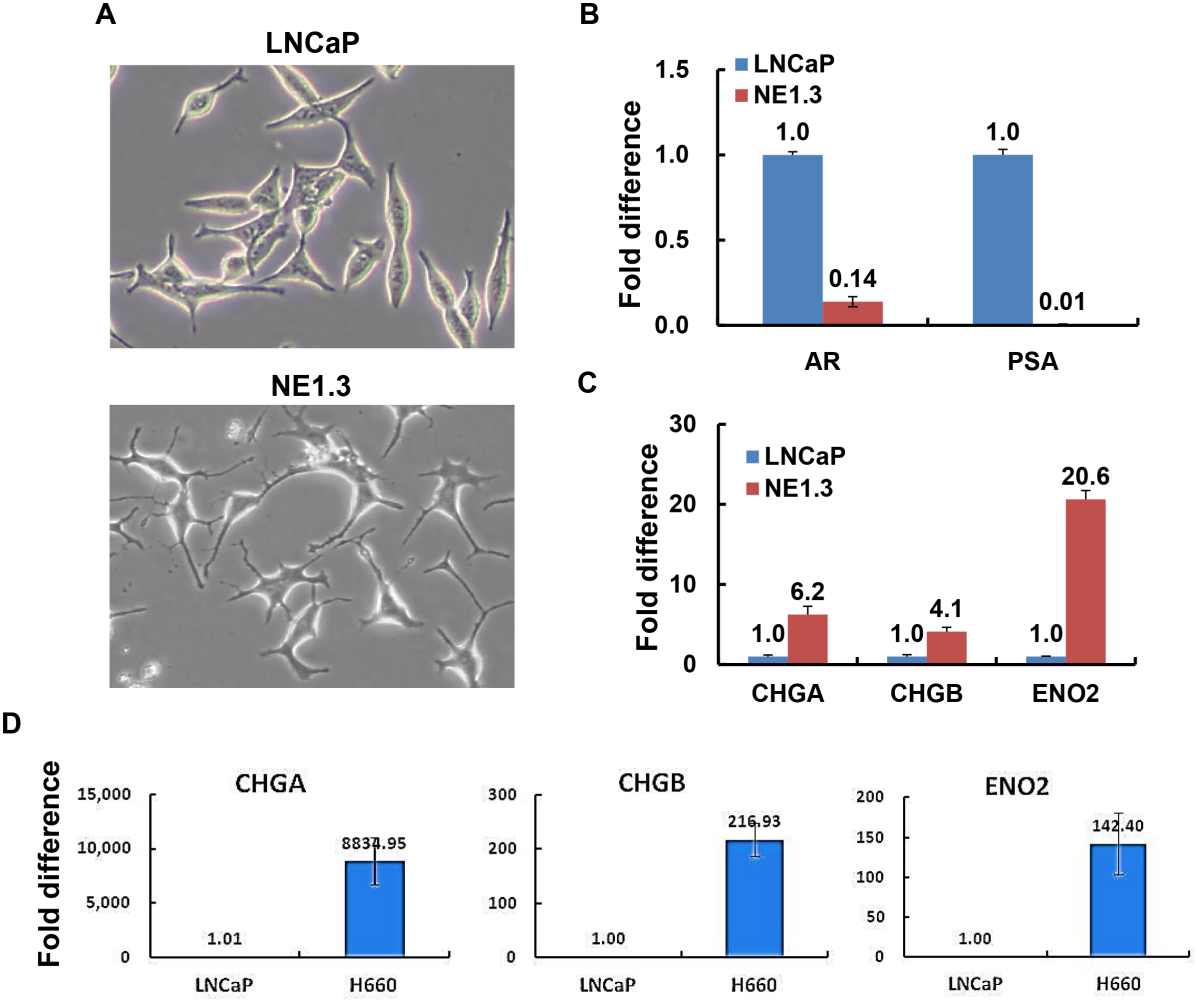
Androgen deprivation treatment (ADT) induces neuroendocrine differentiation in prostate cancer cells **A.** Representative images show that prostate adenocarcinoma cells LNCaP have an epithelial morphology, whereas the ADT-induced, LNCaP-derived neuroendocrine cancer cells NE1.3 show a neuronal morphology with compact, rounded cell bodies and extended, finely branched processes. **B.** RT-PCR shows the expression patterns of androgen receptor (AR) and AR target, prostate specific antigen (PSA), in LNCaP and NE 1.3 cells. Y-axis shows the relative fold differences in expression, normalized to GAPDH. **C, D.** RT-PCR shows the expression patterns of neuroendocrine markers chromogranin A and B (CHGA, CHGB) and enolase 2 (ENO2) in NE1.3 C. and NCI-H660 D. as compared to LNCaP cells. Y-axis shows the relative fold differences in expression, normalized to GAPDH.

To investigate the signaling pathways and molecular mechanisms of neuroendocrine prostate cancer cells, we compared the classic AR-positive adenocarcinoma (PAC) LNCaP cells with NE1.3 cells, neuroendocrine differentiated NEPC cells derived from LNCaP cells through long term androgen deprivation treatment (ADT) [18, 30]. As shown in Figure 1A, LNCaP cells have an epithelial morphology, whereas NE1.3 cells show a neuronal morphology with rounded cell bodies and extended, finely branched processes. NE1.3 cells expressed low levels of AR and AR target PSA, and high levels of NE markers CHGA, CHGB and ENO2 (Figure 1B, 1C). This is consistent with the literature that long term ADT induces NED in PAC cells, mouse models and patients [3, 15, 16, 18, 21, 25, 35, 36, 50]. In addition, we observed that the expression of NE markers was significantly higher in another NEPC cell line NCI-H660 than in PAC cell line LNCaP [51] (Figure 1D).

### GRK3 is up-regulated in ADT-induced NEPC cells

**Figure 2:**
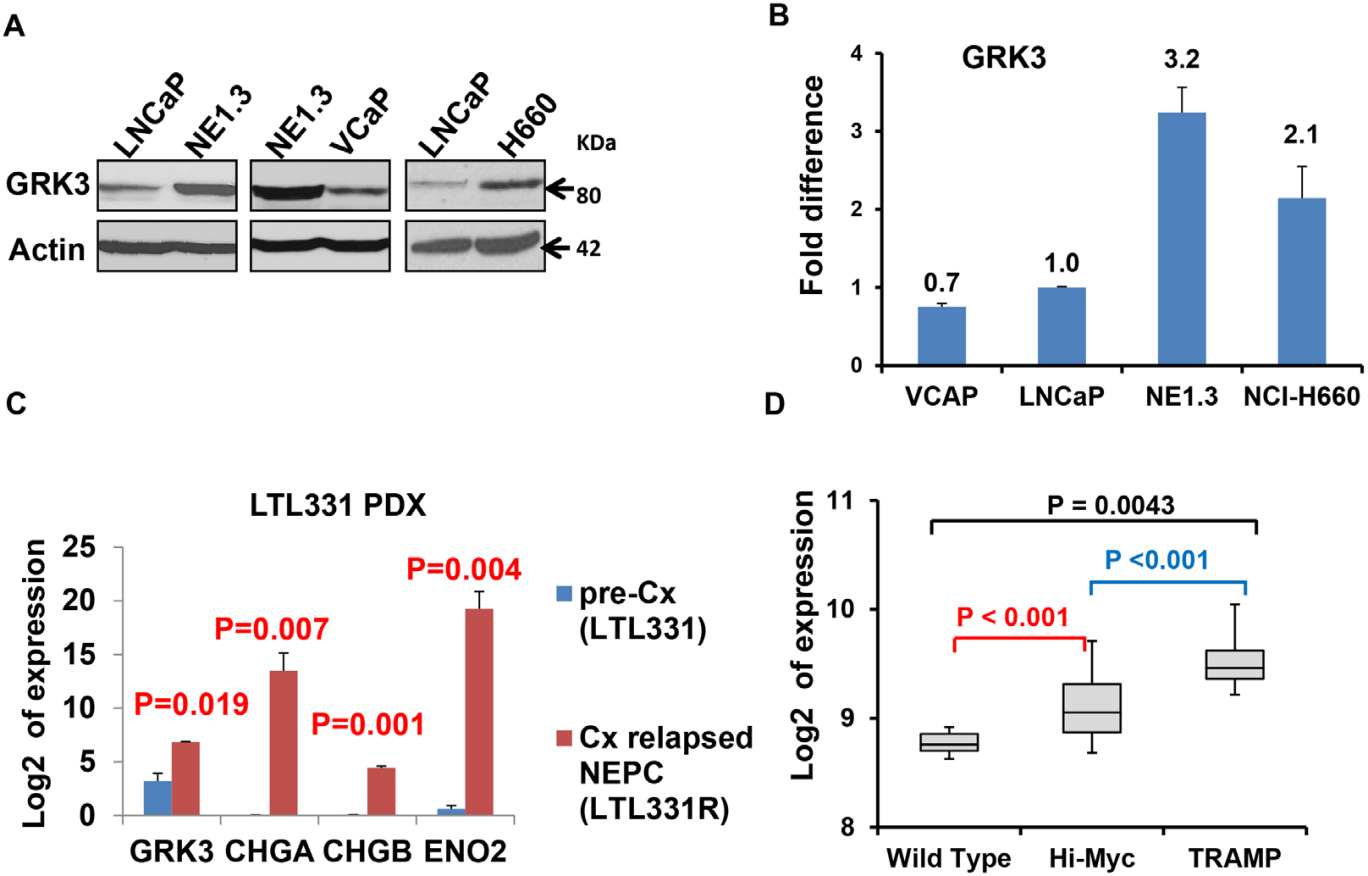
GRK3 is up-regulated in neuroendocrine prostate cancer (NEPC) Western blot assays **A.** and RT-PCR **B.** comparing the expression of GRK3 in prostate adenocarcinoma (PAC) cells LNCaP and VCaP to NEPC cells NE1.3 and NCI-H660. Y-axis: relative fold differences in expression normalized to GAPDH. **C.** GRK3 is significantly up-regulated when the prostate cancer patient derived xenograft (PDX) PAC model LTL331 tumors relapsed after castration and become CRPC/NEPC (LTL331R). RNA-seq data by Akamatsu S et al. [53] were obtained and analyzed for GRK3 expression. Y-axis indicates the log2 of the expression levels. **D.** Expression of GRK3 in different genetically engineered mouse (GEM) models of prostate cancer (GSE53202). TRAMP mice are a classic GEM model for NEPC while Hi-Myc mouse is a classic model of prostate adenocarcinoma (PAC). Y-axis shows the normalized and transformed expression values in the indicated data sets obtained from the GEO database B. and D.

We hypothesized that GRK3 promotes NEPC development. Indeed, we found that GRK3 was significantly up-regulated in NEPC cells NE1.3 and H660 at both mRNA and protein levels as compared with the PAC cells LNCaP and VCaP (Figure 2A, 2B, Supplementary Figure S1A). By analyzing data from a time course study of androgen deprivation of LNCaP cells (GSE8702) [52], we found a similar result, i.e. GRK3 and NE marker ENO2 are up-regulated as the LNCaP cells become androgen-independent after long term ADT (Supplementary Figure S2). To mimic castration-induced neuroendocrine differentiation *in vivo*, we compared the expression of GRK3 and NE markers between untreated PAC prostate cancer patient-deprived xenograft (PDX) LTL331 and NEPC PDX LTL331R that was derived from LTL331 after relapse from castration [14, 32, 33, 53]. GRK3 and NE markers (ENO2, CHGA and CHGB) are significantly up-regulated in LTL331R (Figure 2C).

To further strengthen these results from human prostate cancer cells and PDX models, we investigated GRK3 expression in a classic NEPC genetically engineered mouse (GEM) model, the TRAMP mouse [54, 55]. Analysis of microarray data in GSE58822 and GSE53202 [56, 57] revealed that GRK3 levels were significantly higher in prostate tumors of the TRAMP mice than in normal prostate tissues of wild type mice (P = 0.0043 in GSE58822 and P = 2.17E-16 in GSE53202) (Figure 2D). Interestingly, GRK3 is also expressed more highly in tumors of the TRAMP mice than in a classic GEM model for PAC, the Hi-Myc mice (P = 1.02E-6, GSE53202) (Figure 2D). All together, these results show that GRK3 is induced by ADT and up-regulated in NEPC.

### GRK3 is a direct target of CREB activation that is induced by ADT

**Figure 3:**
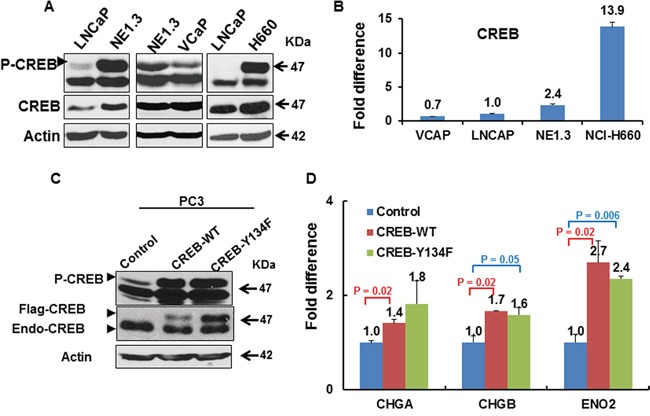
CREB activation is induced by androgen deprivation treatment (ADT) and promotes neuroendocrine differentiation of prostate cancer cells **A.** Western blots show that CREB is up-regulated and hyper-phosphorylated at S133 (activated) in ADT-induced NE1.3 and in NCI-H660 cells, as compared to LNCaP and VCaP cells. **B.** RT-PCR shows elevated expression of CREB in NEPC NE1.3 and NCI-H660 cells compared to LNCaP and VCaP cells. Y-axis shows the relative fold differences in expression, normalized to GAPDH. **C.** A Western blot shows overexpression of flag-tagged wild-type and constitutively active Y134F mutant of CREB. **D.** RT-PCR shows elevated expression of NE markers CHGA, CHGB and ENO2 in the prostate cancer cells overexpressing flag-tagged wild-type or constitutively active Y134F mutant of CREB. Y-axis shows the relative fold differences in expression, normalized to GAPDH.

**Figure 4:**
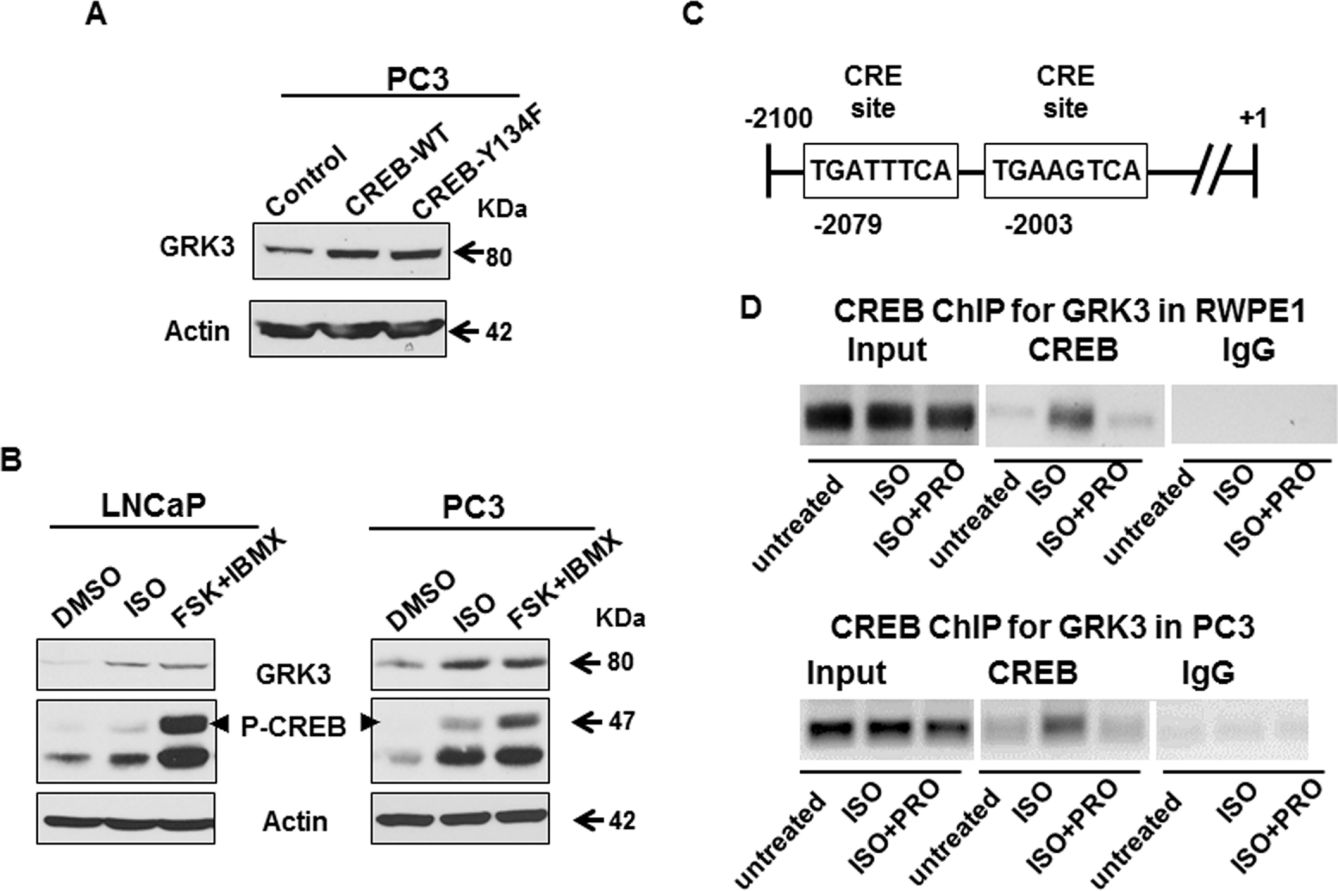
GRK3 is a direct transcriptional target of CREB activation **A.** Western blot shows that GRK3 expression is up-regulated in prostate cancer cells overexpressing CREB-WT and CREB-Y134F cDNAs. **B.** PC3 and LNCaP cells were treated with 10 μM isoproterenol (ISO, beta-adrenergic receptor agonist), or 10 μM forskolin (FSK, adenylyl cyclase activator) + 0.5 mM IBMX (phosphodiesterase inhibitor) for 4 hours. Western blot analysis shows that CREB was hyper-phosphorylated at S133 and GRK3 was significantly up-regulated in both LNCaP and PC3 cells upon treatment with ISO or FSK+IBMX. **C.** Two consensus cAMP response element (CRE) sites, TGANNTCA, are located ~2000 bp upstream of the transcription initiation site in GRK3 promoter. **D.** PC3 and RWPE1 cells were treated with 10 μM ISO or 10 μM ISO+propranolol (PRO, beta-adrenergic receptor antagonist). Chromatin immunoprecipitation (ChIP) was done with anti-CREB and anti-IgG antibodies, followed by PCR using primers designed to recognize the GRK3 promoter sequence around the CRE sites. The ChIP-PCR results were confirmed by DNA gel electrophoresis, using inputs as loading controls. The quantitative measurements of CREB binding to GRK3 promoter are shown in Supplemental Figure S4.

Consistent with reports in literature that CREB activation promotes NED in prostate cancer cells [24, 28, 58], we found that CREB was up-regulated and activated (by pS133 level [59–61]) in NEPC cells NE1.3 and H660 (Figure 3A, 3B, Supplementary Figure S1). Moreover, overexpression of either the CREB wild type cDNA or constitutively active mutant CREB-Y134F cDNA increased the expression of NE markers and GRK3 in prostate cancer cells (Figure 3C, 3D, 4A, Supplementary Figure S1, Supplementary Figure S3A). CREB-Y134F contains a mutation in which tyrosine 134 is changed to phenylalanine, which increases its affinity to PKA, and thus enhances its phosphorylation and activation by PKA [47]. Induction of GRK3 was also observed when we treated prostate cancer cells with beta-adrenergic receptor agonist isoproterenol (ISO), or adenylyl cyclase activator forskolin (FSK) with phosphodiesterase inhibitor IBMX (FSK+IBMX) (Figure 4B, Supplementary Figure S3B). Both treatments are known to activate CREB through PKA phosphorylation at S133 [60–64]. These results support our hypothesis that GRK3 is induced by CREB activation. We further found two consensus cAMP response element (CRE) sites on GRK3 promoter (Figure 4C), which suggests that GRK3 is a direct transcriptional target of CREB activation. To confirm that CREB directly binds to GRK3 promoter, we carried out chromatin immunoprecipitation (ChIP) assay in PC3 and RWPE1 cells. The specific binding of CREB to GRK3 promoter was significantly increased after ISO treatment, and inhibited by beta-adrenergic receptor antagonist propranolol (PRO) [65] (Figure 4D, Supplementary Figure S4). These results indicate that GRK3 is a direct target of CREB activation.

### GRK3 expression positively correlates with CREB and NE markers in human cancer tissues and cell lines

**Figure 5:**
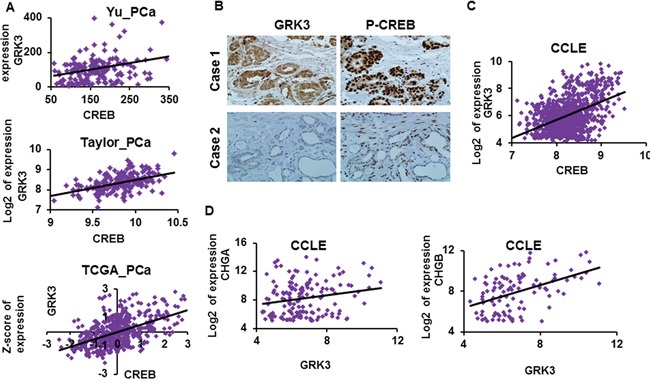
GRK3 expression positively correlates with CREB and NE marker expressions **A.** Results from data mining of published prostate cancer datasets Yu_PCa, Taylor_PCa and TCGA_PCa for mRNA expressions of CREB and GRK3. CREB and GRK3 expressions positively correlate with each other, with Pearson correlation coefficients r = 0.36, 0.44 and 0.52, respectively, and P < 0.000001 in all three datasets. **B.** Immunohistochemistry (IHC) staining was performed on a tissue microarray with 78 cases of human prostate cancer and normal samples using anti-GRK3 and anti p-CREB (S133) antibodies. Staining results in each sample were scored according to the staining area and staining intensity on a scale of 1 to 3. Two representative tumor cases are shown. **C.** GRK3 expression positively correlates with CREB expression in ~1000 human cancer cell lines from the Cancer Cell Line Encyclopedia (Pearson correlation coefficient r = 0.43, P < 0.000001). **D.** GRK3 expression positively correlates with CHGA and CHGB expression in human cancer cell lines from the Cancer Cell Line Encyclopedia (P < 0.00001 in both). Only cell lines with meaningful CHGA or CHGB level (log2 expression >4) were included in this analysis. The normalized and transformed expression values downloaded from GEO database or www.cBioPortal.org were used in our analysis and are plotted on X and Y-axes: normalized expression values for Yu_PCa, Z-scores of expression for TCGA_PCa, log2 transformed expression in all other scatter plots.

Our results reveal that GRK3 is a direct target of CREB, which suggests a positive correlation between CREB and GRK3 expression in human cancer cells and tissues. Indeed, mRNA expression of CREB and GRK3 are positively correlated in several widely cited prostate cancer datasets, such as Yu_PCa [66] (GSE6919), Taylor_PCa [67] (GSE21034) and TCGA_PCa (obtained from www.cBioPortal.org [48, 49]), with Pearson correlation coefficients r = 0.36, 0.44 and 0.52, respectively, and P < 0.000001 for all three (Figure 5A). Furthermore, we carried out analysis of the levels of GRK3 protein expression and CREB activation (by pS133-CREB) in a tissue microarray with 78 cases of human prostate cancer and normal samples. The p-CREB level was found to positively correlate with the expression of GRK3 (Chi Square χ^2^ = 22.2, P = 0.0002) (Figure 5B and Table 1 ). These results support our finding that GRK3 is a target of CREB activation and suggest that the CREB/GRK3 axis is active in human prostate tissues.

**Table 1:** Results of IHC staining of tissue microarrays with 78 cases of human prostate cancer and normal samples

	GRK3		
p-CREB	+ (1)	++ (2)	+++ (3)
+ (1)	22	7	1
++ (2)	7	9	5
+++ (3)	4	14	9

Chi-square test showed that p-CREB levels positively correlate with the expression of GRK3 (Chi Square χ^2^ = 22.2, P = 0.0002)

To determine if the positive correlation between CREB and GRK3 exists beyond prostate cancer, we analyzed their expression patterns in ~1000 human cancer cell lines from the Cancer Cell Line Encyclopedia (CCLE) [68]. GRK3 expression indeed positively correlates with CREB expression (Pearson correlation coefficient r = 0.43, P < 0.000001) (Figure 5C). To test whether GRK3 expression correlates with the expression of NE markers as well, we analyzed the CCLE cancer cell lines with measurable CHGA or CHGB expression (log2 transformed expression >4) and found that GRK3 expression positively correlates with CHGA (r = 0.41, P < 0.00001) and CHGB expressions (r = 0.24, P < 0.00001) (Figure 5D). These results suggest that positive correlation between GRK3 and NED markers exists broadly in human cancer cell lines.

### GRK3 is a critical activator for NE phenotypes of prostate cancer cells

**Figure 6:**
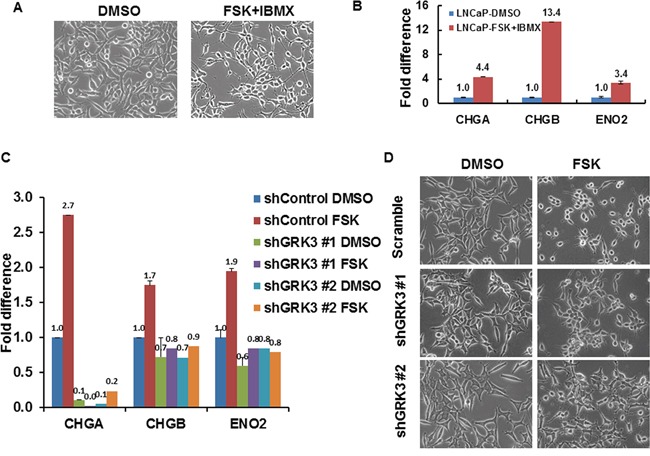
GRK3 is critical for CREB induced neuroendocrine differentiation of prostate cancer cells **A.** LNCaP cells exhibited a typical epithelial, fusiform morphology, tapering into unbranched processes typically less than one cell body length, whereas FSK+IBMX treatment (10 μM FSK + 0.5 mM IBMX) treatment resulted in a neuronal morphology with compact, rounded cell bodies and extended numerous long, fine, branched processes with defined growth cones. **B.** RT-PCR comparing the expressions of NE markers CHGA, CHGB and ENO2 in the mock or FSK+IBMX treated LNCaP cells. Y-axis shows the relative fold differences in expression, normalized to GAPDH. **C.** RT-PCR results show that the expressions of CHGA, CHGB and ENO2 could not be up-regulated with FSK+IBMX upon GRK3 down-regulation in LNCaP cells. Y-axis shows the relative fold changes in expression, normalized to GAPDH. **D.** Representative images of LNCaP cells that express either Scramble control shRNA or two specific GRK3 shRNAs after treatment with either DMSO vehicle or forskolin (FSK, 10uM) for 4 hours. The GRK3 knockdown efficiency in LNCaP-shGRK3 cells is shown in Supplemental Figure S6A.

Upon showing that GRK3 is up-regulated in NEPC as a direct target of CREB activation, we next investigated whether GRK3 plays a critical role in promoting NED, induced by ADT or CREB activation. Consistent with the literature [20, 22], LNCaP cells developed features of neuronal morphology upon CREB activation by FSK+IBMX treatment (Figure 6A). As expected, the treatment also significantly increased expressions of NE markers CHGA, CHGB and ENO2 (Figure 6B, Supplementary Figure S5). We simultaneously carried out the same FSK+IBMX treatment on LNCaP cells expressing GRK3 shRNA# 1 or shRNA# 2 (Figure 6C, 6D). Notably, FSK+IBMX could no longer induce the expression of NE markers and obvious NE morphology, upon GRK3 knockdown (Figure 6C, 6D, supplemental Figure S6A). These results indicate that GRK3 is required for NED induction by CREB activation in LNCaP cells.

**Figure 7:**
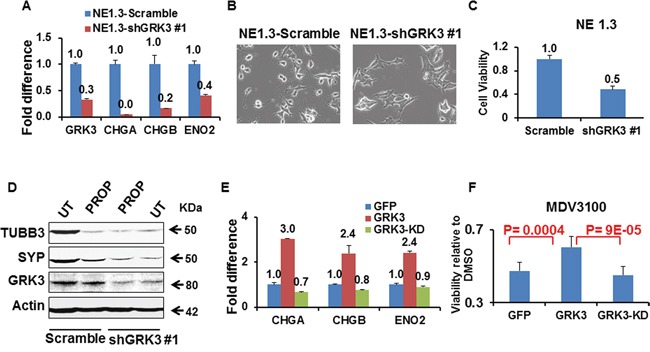
GRK3 controls neuroendocrine phenotypes of prostate cancer cells **A.** RT-PCR comparing the expressions of NE markers CHGA, CHGB and ENO2 in NE1.3 cells upon GRK3 down-regulation. Y-axis shows the relative fold differences in expression, normalized to GAPDH. **B.** Representative pictures of NE1.3 cells expressing either Scramble control shRNA or GRK3 shRNA. Upon GRK3 down-regulation, the neuronal morphology of the NE1.3 cells (such as the compact, rounded cell bodies and extended and branched processes) disappeared. **C.** NE1.3 cells with and without GRK3 down-regulation were seeded in 96 well plates and were allowed to grow for 72 hours (6 replicates). The cell numbers were measured using Alamar Blue^®^ cell viability assay and the fold difference is plotted on the Y-axis. **D.** NE1.3 cells expressing Scramble or shGKR3 were either untreated (UT) or treated with beta-adrenergic receptor antagonist, propranolol (PROP, dissolved in H_2_O, 10 μM, 4 hours), followed by western blotting analysis for expression of NE markers synaptophysin (SYP) and tubulin-beta III (TUBB3) [81, 82]. **E.** RT-PCR comparing the expression of NE markers in LNCaP cells upon overexpression of GFP, GRK3 (wild-type) or GRK3-KD (kinase dead) cDNA. Y-axis shows the relative fold differences in expression, normalized to GAPDH. **F.** LNCaP-GFP, LNCaP-GRK3 and LNCaP-GRK3-KD cells were treated with DMSO vehicle or 5uM MDV3100 (enzalutamide) for 10 days. The cell numbers were measured using Alamar Blue^®^ cell viability assay. The fold difference in viability in MDV3100 relative to DMSO for each cell lines are plotted on the Y-axis. P values were calculated using Student t-test, based on data from eight replicates. The GRK3 knockdown in NE1.3-shGRK3 cells and GRK3 overexpressing in LNCaP-GRK3 cells are shown in Supplemental Figure S6B and S6C.

To evaluate whether GRK3 suppression is sufficient to reverse ADT-induced NED, we down-regulated GRK3 expression in ADT-induced NEPC cells NE1.3 using GRK3 specific shRNA [37] (Supplemental Figure S6B). As shown in Figure 7A, the expression of NE markers CHGA, CHGB and ENO2 was reduced upon GRK3 down-regulation. In addition, the neuronal morphology – small and rounded cell bodies, and extended, fine branches – disappeared (Figure 7B). Notably, GRK3 knockdown inhibited the proliferation of NE1.3 cells (Figure 7C).

To further establish an essential role of the CREB-GRK3 axis in NED of prostate cancer cells, we next tested the hypothesis that upon GRK3 knockdown in NEPC cells, inhibition of CREB cannot further repress the expression of NE markers. Results from propranolol (PROP), an inhibitor of beta-adrenergic signaling and CREB activation, provide evidences supporting this hypothesis (Figure 7D). This result, together with the data in Figure 6C-D, indicates that GRK3 is a key mediator of CREB activation in promoting NED of prostate cancer cells.

Finally, to directly examine a causal role of GRK3 in NED of prostate cancer cells, we overexpressed GRK3 wild type (WT) cDNA and kinase dead (KD) cDNA with a K220R mutation [37, 69] in LNCaP cells (Supplemental Figure 6C). GRK3-WT induced the expression of NE markers CHGA, CHGB, and ENO2 but GRK3-KD did not, which suggests that the GRK3 kinase activity is required for its induction of NE markers (Figure 7E). Importantly, expressing GRK3-WT cDNA rendered LNCaP cells more resistant to MDV3100 than expressing GFP control or GRK3-KD cDNA (Figure 7F). In addition, overexpressing GRK3 promoted LNCaP cell-derived primary tumor growth *in vivo* [37]. Collectively, these results indicate that GRK3 is a critical activator of NE phenotypes, ADT resistance, and prostate cancer progression.

## DISCUSSION

In this study, we have demonstrated that GRK3 is induced by androgen deprivation treatment (ADT) as a direct target of ADT-activated CREB, and that expression of GRK3 positively correlates with expression and activity of CREB in prostate cancer cells and tissues. Of note, overexpression of GRK3 is sufficient to promote neuroendocrine differentiation (NED) and resistance to MDV3100, while GRK3 silencing blocks CREB-induced NED, reverses NE phenotypes, and inhibits proliferation of NEPC cells. These results suggest that ADT activates a critical signaling pathway, the CREB/GRK3 axis, in promoting NED of prostate cancer cells.

Previously, GRK3 was shown to be up-regulated by chronic treatments with adrenaline or corticotropin release factor (CRF1) [70, 71]. These two stress hormones signal to their respective GPCRs, beta-adrenergic receptors and CRF1 receptor, that couple to Gαs protein to raise cAMP levels in cells [70, 71]. It was unclear how the stress hormones induce GRK3 expression. Our results reveal that GRK3 is a direct target of activated CREB, which provides a plausible explanation for GRK3 induction by the stress hormones.

Moreover, our results introduce a new paradigm of CREB/GRK3 signaling. Since a perceived role of GRK3 is to phosphorylate and desensitize CRF1 receptor that signals to activate CREB, up-regulation of GRK3 by CRF1 was hypothesized as a negative feedback regulation to control the activation of the receptor [71]. In CREB-induced NED of prostate cancer cells, we have demonstrated, for the first time, that GRK3 can be a downstream mediator of CREB activation. Furthermore, our results indicate a positive correlation of basal expression of CREB and GRK3 in human prostate cancer, normal tissues, and a broad range of human cell lines of many cancer types (CCLE) (Figure 5). Therefore, it can be speculated that the CREB/GRK3 axis may be active in wide range of cancer cells and biological contexts.

The data presented in our previous [37] and current studies suggest that targeting GRK3 may be a viable approach to inhibit prostate cancer progression and NEPC development. Kinases are known to be druggable [72, 73] and several kinase inhibitors have been approved as cancer therapeutics [74–77]. We have presented several lines of evidence suggesting that kinase activity of GRK3 is critical for its roles in cancer. Unlike the GRK3 wild type form, the GRK3-kinase dead form can no longer repress two established tumor suppressors thrombospondin 1 (TSP1) and plasminogen activator inhibitor 2 (PAI2) [37]. GRK3-kinase dead form was also incapable to induce NE marker expression in prostate cancer cells (Figure 7E). These results support the rationale to identify GRK3 kinase inhibitors as candidates for new cancer drugs.

GRK3 may control NE phenotypes through its regulation of some specific GPCRs in NEPC. Alternatively, GRK3 may act through phosphorylating non-GPCR substrates or function as a scaffold protein, as having been shown for other GRKs, such as GRK2 and GRK5 [78–80]. Further characterization of these two potential mechanisms of action for GRK3 in NEPC will be helpful in designing drugs for this target.

Taken together, our results demonstrate that GRK3 is a new activator for neuroendocrine phenotypes and ADT resistance in prostate cancer cells. It is a direct target and a critical mediator of activated CREB in promoting NE differentiation. These results expand our knowledge of NEPC development, prostate cancer progression, and GRK3 as a prospective novel drug target for aggressive prostate cancers.

## MATERIALS AND METHODS

### Cell culture

Human prostate cancer cells LNCaP and PC3 were maintained in regular RPMI 1640 medium supplemented with 10% FBS (Gibco), 1% glutamine, 1% penicillin and streptomycin. ADT-induced NEPC cells NE1.3 were maintained in phenol red-free RPMI 1640 medium supplemented with 5% charcoal-striped FBS (Gibco) and 1% penicillin and streptomycin [18]. Prostate neuroendocrine small cell carcinoma cells NCI-H660 were obtained from ATCC and cultured according to ATCC guidance. VCaP cells were maintained in the DMEM medium supplemented with 10% FBS (Gibco), 1% glutamine, 1% penicillin and streptomycin. RWPE-1 cells were grown in keratinocyte serum free medium with 0.05 mg/ml BPE and 5 ng/ml EGF, according to ATCC guidance. Cultures were grown in a 37°C incubator with 5% CO2.

### cDNA/shRNA transduction and transfection in prostate cancer cells

LNCaP cells expressing GFP, GRK3 (wild type cDNA) or GRK3-KD (kinase dead cDNA) were generated through retroviral transduction as previously described [37]. Preparation and usage of shGRK3 lentivirus have also been described [37]. LNCaP and NE1.3 cells were infected by shGRK3 lentivirus at MOI of 5 in the presence of polybrene(8 μg/ml). shScramble was used as control. PC3 cells were transfected with the mammalian expression vector pcDNA3.1, Flag-pcDNA3.1-CREB (wild type), Flag-pcDNA3.1-CREB (Y134F) [47] (kindly provided by Dr. Rebecca Berdeaux at UT-Houston) using TransIT-LT1 transfection reagent (Mirus, Madison, WI, USA). Transfected PC3 cells were selected with 400μg/ml of G418.

### Reverse transcription and quantitative real-time PCR

Total RNA was extracted from the indicated cells by using TRIzol Reagent (Life technology). The RNA concentration and purity were measured by NanoDrop 2000 UV-vis Spectrophotometer (Thermo Scientific, USA). 3μg of total RNA was used to generate cDNA using the iScript Reverse Transcription Supermix (Bio-Rad, USA). Real time PCR was performed using SsoFast EvaGreen Supermix in CFX96 Thermal Cycler (Bio-Rad, USA). PCR-based amplification was performed using the following primers. GRK3 forward, 5′-gcagtgccgactggttct-3′, GRK3 reverse, 5′-gtctgaaagggctgtgacct-3′; CREB forward, 5′-ggagcttgtaccaccggtaa-3′, CREB reverse, 5′-gcatctccactctgctggtt-3′;CHGA forward, 5′-tacaaggagatccggaaagg-3′, CHGA reverse, 5′-ccatctcctcctcctcctct-3′; CHGB forward, 5′-cacgccattctgagaagagc-3′, CHGB reverse, 5′-tctcctggctcttcaaggtg-3′; ENO2 forward, 5′-ctgtggtggagcaagagaaa-3′, ENO2 reverse, 5′-acacccaggatggcattg-3′; AR forward, 5′-gccttgctctctagcctcaa-3′, AR reverse, 5′-ggtcgtccacgtgtaagttg-3′; PSA forward, 5′-cacagcctgtttcatcctga-3′, PSA reverse, 5′-atatcgtagagcgggtgtgg-3′; GAPDH forward, 5′-agccacatcgctcagacac-3′, GAPDH reverse, 5′-gcccaatacgaccaaatcc-3′. For all RT-PCR analysis, GAPDH was used to normalize RNA input and expression levels were calculated according to the comparative C_T_ method (ΔΔC_T_).

### Western blot

Cells were washed in ice-cold PBS and lysed in the lysis buffer (30 mM Tris, 200 mM NaCl, 1.5 mM MgCL2, 0.4 mM EDTA, 20% Glycerol, 1% NP-40, 1 mM DTT) with Complete Mini protease inhibitor cocktail and PhosSTOP phosphatase inhibitor cocktail (Roche Applied Science). The protein concentration of cell lysates was determined using Pierce BCA Protein Assay Kit (Thermo Scientific, USA). Equal amounts of cell lysates were separated by SDS-PAGE (SDS-polyacrylamide gel electrophoresis) and transferred onto a polyvinylidene difluoride (PVDF) membrane (Bio-Rad). The membranes were blocked with TBST (50mM Tris-HCI, pH8.0, 100mM NaCl and 0.1% Tween 20) containing 5% nonfat dried milk or BSA for 1 h at room temperature, and then probed with the indicated primary antibodies overnight at 4°C. The primary antibodies used were as follows: Anti-GRK3 (Epitomics, USA), Anti-CREB (Cell signaling, USA), Anti-p-CREB (Cell signaling, USA), Anti-Actin (Santa Cruz, USA). After washes, the membranes were incubated with HRP-conjugated secondary anti-mouse or anti-rabbit antibodies (Cell Signaling Technology) for 1 h at room temperature. Finally, the immunoreactive bands were developed with Pierce ECL Western Blotting Substrate (Thermo Scientific, USA) on Blue Basic autoradiography Film (Bioexpress).

### Chromatin immunoprecipitation assay (CHIP)

Cells were cross-linked in 1% formaldehyde for 10 min at room temperature, and then quenched with glycine. Cells were washed twice with 1×PBS and lysed in SDS Lysis Buffer (1% SDS, 10 mM EDTA, 50 mM Tris-HCl, pH 8.1 and freshly added protease/phosphatase inhibitors), then sonicated on ice, for 12 cycles of 20 sec ON and 40 sec OFF at 40% amplitude using Branson Low Power Ultrasonic Systems 2000 LPt/LPesonicator (Fisher Scientific, Pittsburgh, PA). The supernatants were collected by centrifugation at 14,000 rpm for 10 min at 4°C. 50 μl of supernatant was diluted in 450 μl dilution buffer (1% Triton X-100, 2 mM EDTA, 20 mM Tris-HCl pH 8.1, 150 mM NaCl supplemented with 0.1% NP40, protease and phosphatase inhibitors). Samples were precleared with 20 μl of protein A/G-agarose beads for 2 h at 4°C. At this stage, 20μl of supernatant was kept as input. Immunoprecipitations were performed overnight with 2 μg anti-p-CREB (Millipore) or anti-IgG antibody. The immune complexes were captured by incubation with 20 μl of protein A/G-agarose beads for 2 hour at 4°C. The immunoprecipitates were washed sequentially for 5 minutes each at 4°C in low salt buffer with150 mM NaCl, high salt buffer with 500 mM NaCl, LiCl buffer with 250 mM LiCl and finally the TE buffer. Beads were then eluted with 100 μl of Elution Buffer (1% SDS, 100 mM NaHCO3). Proteinase K and 0.2 M NaCl were used to reverse the crosslinking. DNA was then extracted by phenol-chloroform extraction. 5 μl of DNA was subjected to real time PCR. Primers used to measure the enrichment of GRK3 promoter DNA sequence containing the CRE are: forward (5′-GCCTCTAAGATCACCCAGCA-3′), and reverse(5′-AGACCTGACATCTGCCTACA-3′). The enrichment of ChIP DNA was calculated as percentage of input. The PCR products were resolved electrophoretically on a 2% agarose gel and visualized by ethidium bromide staining.

### Immunohistochemistry staining on human prostate tumor tissue microarray

The Universal Elite ABC kit (Vector Labs) was used for immunohistochemistry (IHC) staining, according the manufacturer's instructions. Briefly, slides of five micrometer sections from 78 cases of formalin-fixed and paraffin-embedded prostate cancer and normal tissue blocks were dewaxed in 60°C oven for 2 hours and rehydrated through incubating in xylene and alcohol series. Antigen retrieval was done in 10 mmol/L sodium citrate buffer (pH 6.0) in a food steamer for 30 minutes. After suppressing the endogenous peroxidase activity the sections were incubated in normal horse serum to prevent nonspecific immunoglobulin binding. Upon PBS wash, the sections were then treated with the anti-human p-CREB (Cell signaling, USA) or anti-human GRK3 antibody (Epitomics, USA) at 4°C overnight. A streptavidin-HRP detection system was used to reveal specific binding. Immunoreactivity was scored as following: staining intensity −/+, <25% positive cells (weak, score 1); staining intensity ++, 25–50% positive cells (intermediate, score 2); and staining intensity +++, >50% positive cells (strong, score 3). Percent of positive cells and staining intensity were scored independently by two experienced researchers.

### Microarray data mining

The indicated GSE microarray data sets were downloaded from the GEO database http://www.ncbi.nlm.nih.gov/gds. The TCGA_PCa data on CREB and GRK3 were downloaded from http://www.cbioportal.org [48, 49]. The normalized and transformed gene expression values from the sources were used in our analysis and statistical calculation.

### Cell proliferation assay

AlamarBlue® cell viability reagent (Thermo Fisher Scientific) was used to estimate the cell numbers and the cell proliferation assay was performed according to the manufacturer's protocol. Briefly, 1000 cells were seeded in each well of 96 cell well plates and were allowed to proliferate for 72 hours in regular culture media and conditions. AlamarBlue® cell viability reagent was added to the cells and incubated at 37°C for 1–4 hours. Infinite® M1000 spectrophotometer was used (Tecan US, Inc. Morrisville, NC) to read fluorescence at excitation wavelength 535 nm with emission wavelength at 595 nm. The readings were plotted with Y-axis showing the relative cell number.

### MDV3100 treatment

LNCaP-GFP, -GRK3 and GRK3-KD cells were seeded in 24 well plates (4000 cells per well, six replicates per cell line, per treatment). Cells were treated with vehicle or 5 μM MDV3100 for 10 days. Fresh media and drugs were replenished after 5 days of treatment. Cell viability was studied with the AlamarBlue® cell viability reagent (Thermo Fisher Scientific) as described above.

## SUPPLEMENTARY FIGURES


