# Supplementary material for: GRK3 is a direct target of CREB activation and regulates neuroendocrine differentiation of prostate cancer cells
Source: Oncotarget. 2016 May 14;7(29):45171–85. doi: 10.18632/oncotarget.9359 (PMC5216714; doi:10.18632/oncotarget.9359)
Supplement: Supplementary file 1 [file oncotarget-07-45171-s001.pdf]

SUPPLEMENTARY FIGURES

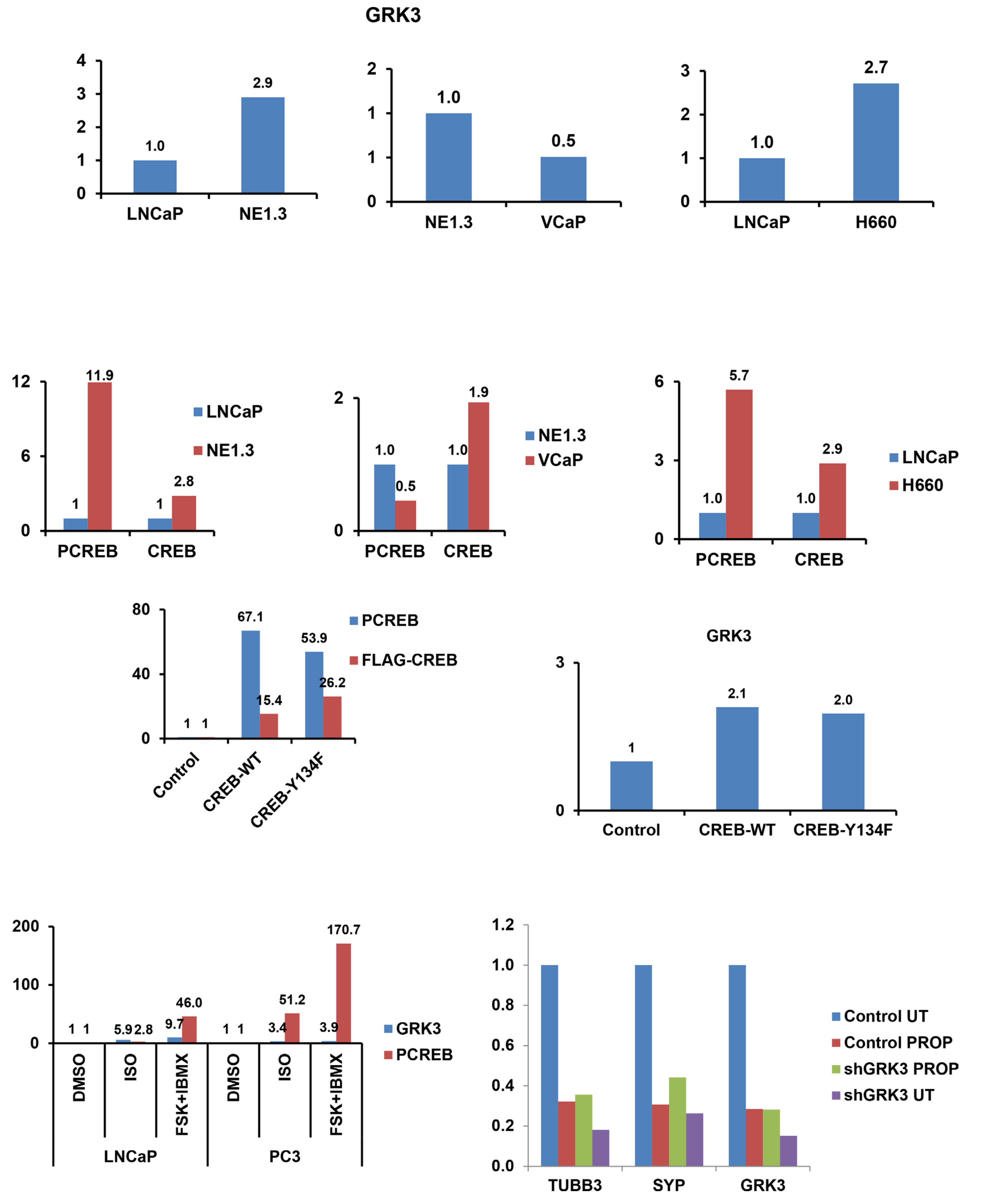

Supplementary Figure S1: Quantification of all the western blots. Y-axes show the fold differences of protein expression normalized to actin for each protein in each western blot.

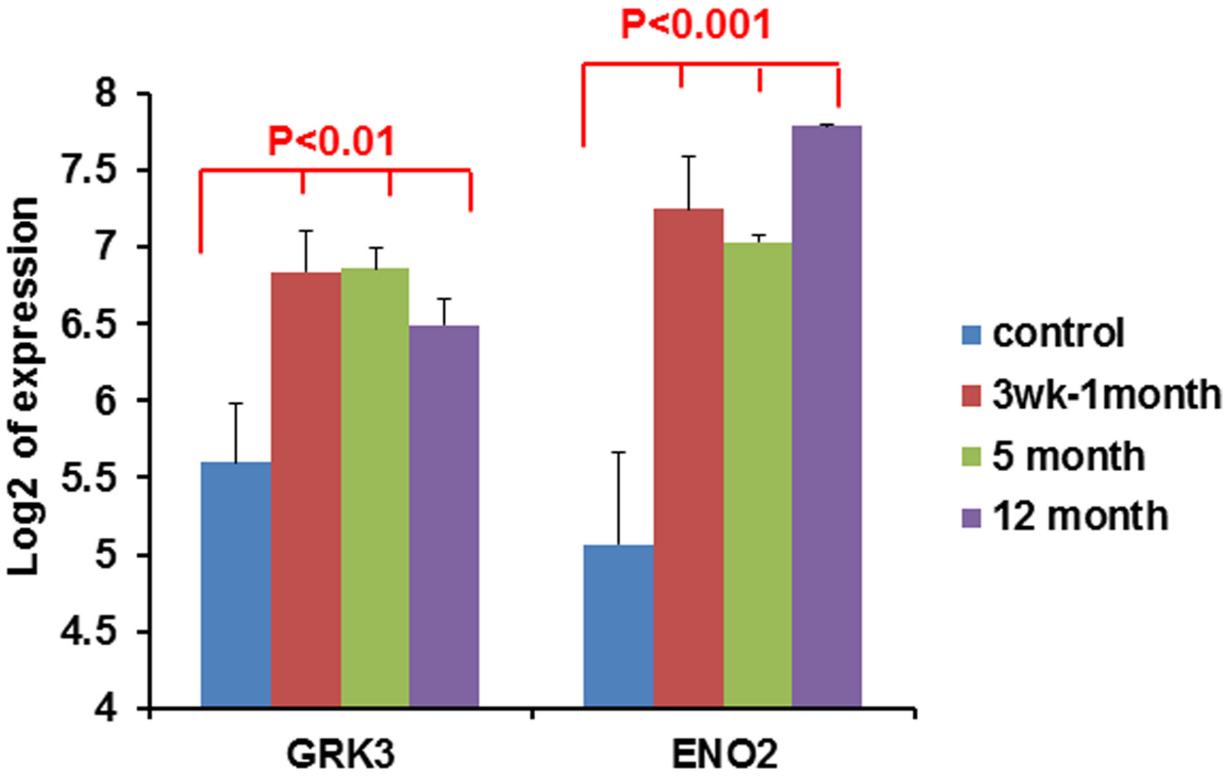

Supplementary Figure S2: Results from data mining of a time course study of androgen deprivation of LNCaP cells (GSE8702). Y axis shows the log2 transformed expression of GRK3 and NE marker ENO2 at different durations of androgen deprivation of LNCaP cells (untreated; early, 3 week-1 month; midterm, 5 months; late, 12 months).

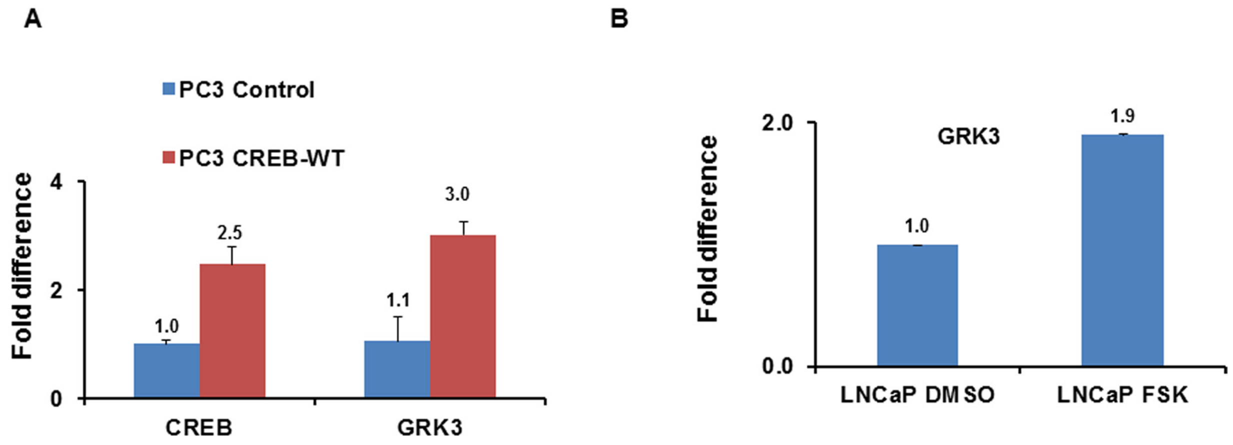

Supplementary Figure S3: A. RT-PCRs showing the expressions of CREB and GRK3 in PC3 cells overexpressing CREB cDNA. GRK3 expression is up-regulated in cells overexpressing CREB. **B.** PC3 cells were treated with CREB activator forskolin (FSK, 10 μM, 4 hours). GRK3 expression was up-regulated in cells treated with FSK. Y-axis shows the relative fold differences in expression, normalized to GAPDH.

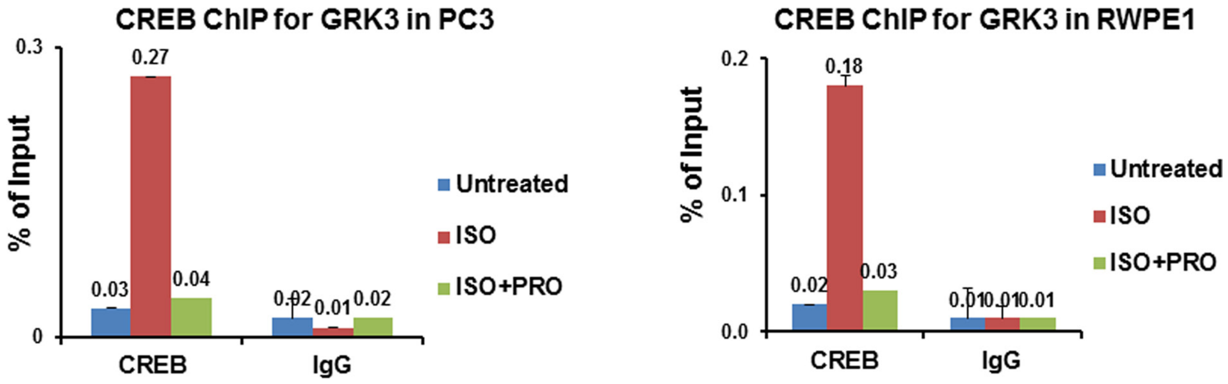

Supplementary Figure S4: The ChIP-PCR results were quantified for Figure 4D as % binding of the input and plotted on the Y-axis. PC3 and RWPE1 cells were treated with 10 µM ISO (isoproterenol, beta-adrenergic activator) or 10 µM ISO + 10 µM PRO (propranolol, beta-adrenergic receptor antagonist). Chromatin immunoprecipitation (ChIP) was done with anti-CREB and anti-IgG antibodies, followed by PCR using primers designed to recognize the GRK3 promoter sequence around CRE sites.

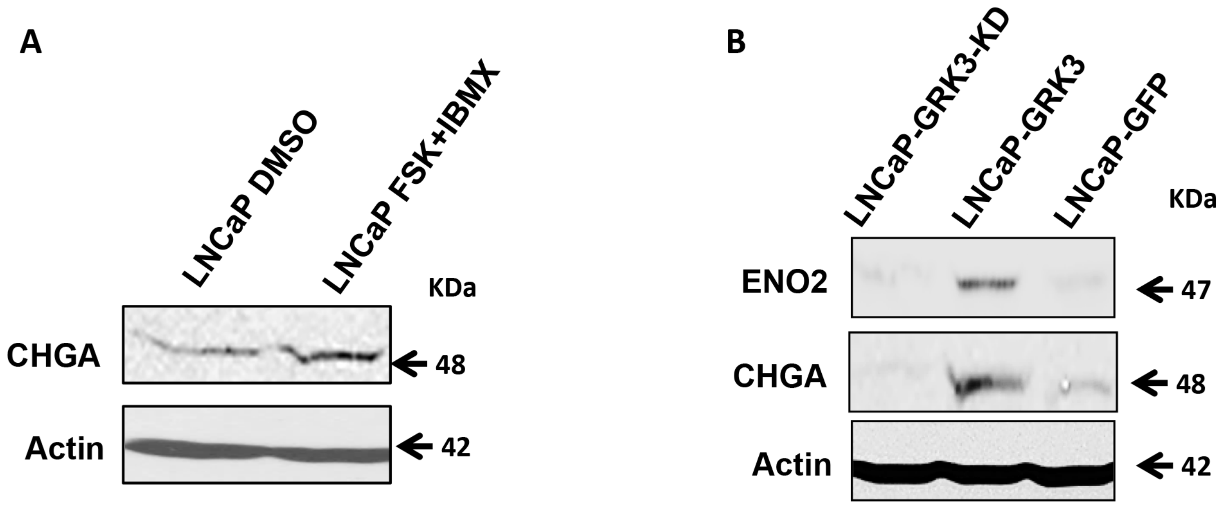

Supplementary Figure S5: A. LNCaP cells were treated with CREB activator forskolin (FSK, 10 μM, 4 hours) and 0.5 mM IBMX (phosphodiesterase inhibitor, 4 hours). A western blot shows that treatment with forskolin and IBMX (FSK+IBMX) results in the higher expression of NE marker CHGA. **B.** Overexpression of GRK3, but not its kinase-dead mutant GRK3-KD, results in the up-regulation of NE markers CHGA and ENO2 in LNCaP cells.

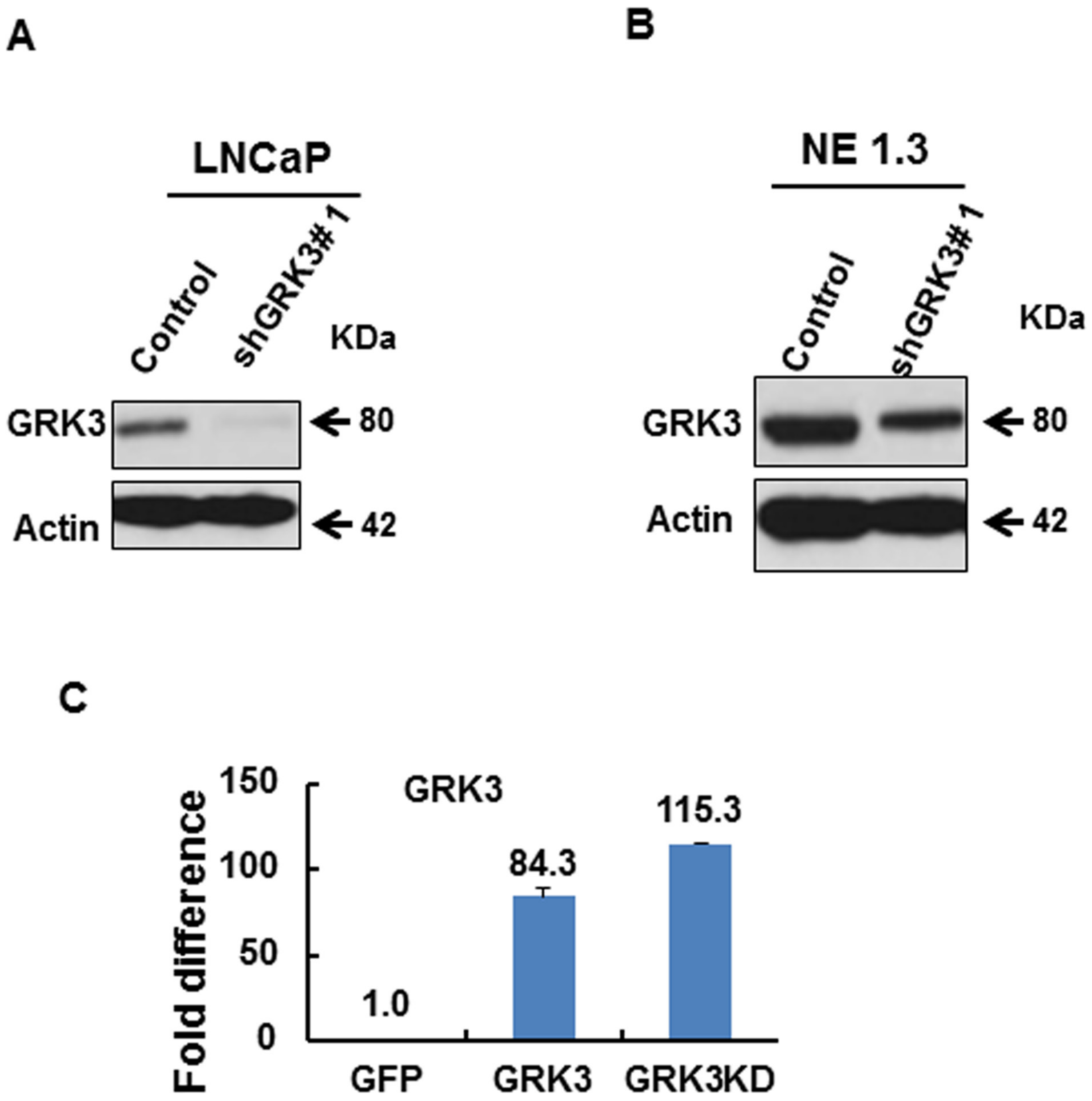

Supplementary Figure S6: A–B. Western blots confirming the down-regulation of GRK3 in LNCaP-shGRK3 and NE1.3-shGRK3 cells. **C.** RT-PCR confirming the overexpression of GRK3 in LNCaP-GRK3 and LNCaP-GRK3 KD cells.
